# Identification of an Autophagy-Related Prognostic Signature for Clear Cell Renal Cell Carcinoma

**DOI:** 10.3389/fonc.2020.00873

**Published:** 2020-05-29

**Authors:** Mei Chen, Shufang Zhang, Zhenyu Nie, Xiaohong Wen, Yuanhui Gao

**Affiliations:** Central Laboratory, Affiliated Haikou Hospital of Xiangya Medical College, Central South University, Haikou, China

**Keywords:** autophagy, clear cell renal cell carcinoma, prognosis, the cancer genome atlas, platinum drug resistance

## Abstract

Abnormal autophagy is closely related to the development of cancer. Many studies have demonstrated that autophagy plays an important role in biological function in clear cell renal cell carcinoma (ccRCC). This study aimed to construct a prognostic signature for ccRCC based on autophagy-related genes (ARGs) to predict the prognosis of ccRCC. Differentially expressed ARGs were obtained from ccRCC RNA-seq data in The Cancer Genome Atlas (TCGA) database. ARGs were enriched by gene ontology (GO) and Kyoto Encyclopedia of Genes and Genomes (KEGG). The prognostic ARGs used to construct the risk score models for overall survival (OS) and disease-free survival (DFS) were identified by Cox regression analyses. According to the median value of the risk score, patients were divided into a high-risk group and a low-risk group. The OS and DFS were analyzed by the Kaplan-Meier method. The predictive accuracy was determined by a receiver operating characteristic (ROC) curve analysis. Additionally, we performed stratification analyses based on different clinical variables and evaluated the correlation between the risk score and the clinical variables. The differentially expressed ARGs were mainly enriched in the platinum drug resistance pathway. The prognostic signatures based on 11 ARGs for OS and 5 ARGs for DFS were constructed and showed that the survive time was significantly shorter in the high-risk group than in the low-risk group (*P* < 0.001). The ROC curve for OS exhibited good predictive accuracy, with an area under the curve value of 0.738. In the stratification analyses, the OS time of the high-risk group was shorter than that of the low-risk group stratified by different clinical variables. In conclusion, an autophagy-related signature for OS we constructed can independently predict the prognosis of ccRCC patient, and provide a deep understanding of the potential biological mechanisms of autophagy in ccRCC.

## Introduction

Renal cell carcinoma (RCC) is a malignant tumor originating from the renal tubular epithelium. It is a common malignant tumor of the urinary system, and clear cell renal cell carcinoma (ccRCC) is the most common subtype (1). Currently, surgical resection is the main treatment for ccRCC, but ccRCC has a poor prognosis and is likely to recur (2). Common clinical variables, such as the TNM stage, have good prognostic value (3). However, because of tumor heterogeneity the TNM stage cannot accurately predict the prognosis of patients (4). Therefore, the discovery of new molecular targets in ccRCC is the first requirement for achieving early diagnosis and improving the survival rate of ccRCC patients.

Autophagy is a highly conserved intracellular self-digestion process that maintains cellular homeostasis through lysosomes (5). Autophagy plays a key role in maintaining the balance between the synthesis and degradation of cell components (6). Dysregulation of autophagy is closely related to cancer (7). Initially, autophagy can prevent or delay tumor formation, but once tumors are formed, autophagy can promote tumor progression and protect cancer cells from environmental damage (8).

Many studies have demonstrated the role of autophagy in ccRCC. Autophagy is a therapeutic target for renal cancer (9, 10). Studies have shown that promoting autophagy can inhibit the progression of ccRCC (11, 12). The lower the level of autophagy is, the higher the stage and grade of ccRCC (13). Many drugs have been developed to promote autophagy in renal carcinoma and have achieved good therapeutic effects (14). However, these studies mainly focused on the influence of autophagy on the progression and treatment of ccRCC, and few researchers have studied the role of autophagy in the prognosis of ccRCC.

In this study, 45 differentially expressed autophagy-related genes (ARGs) were obtained from the expression data of patients in the kidney clear cell carcinoma (KIRC) cohort in The Cancer Genome Atlas (TCGA) database, and the biological functions of these differentially expressed ARGs were analyzed. These analyses provided further insight into the roles of these ARGs in ccRCC. Importantly, we constructed risk score models based on 11 prognostic ARGs for overall survival (OS) and five prognostic ARGs for disease-free survival (DFS) and found that the autophagy-related signature can independently predict the prognosis of ccRCC patients without considering clinical variables, suggesting that those autophagy-related signatures are reliable prognostic marker in ccRCC patients.

## Materials and Methods

### Data Sources

We obtained 232 ARGs from the HADb (Human Autophagy Database, http://www.autophagy.lu/) and then downloaded the FPKM-standardized RNA-seq data and the clinical and OS information from the KIRC cohort in the TCGA database (https://portal.gdc.cancer.gov/). Table 1 shows the basic clinical characteristics of the patients with ccRCC in the TCGA database. We obtained the DFS data of 431 ccRCC patients from cBioportal (https://www.cbioportal.org/). A total of 222 ARGs with expression values were obtained.

**Table 1:** Clinical characteristics of ccRCC patients in the TCGA database.

**Characteristics**		**Total**	**%**
Age at diagnosis (y)		58 (26~90)	
Gender	Male	346	64.43
	Female	191	35.57
Grade	G1	14	2.65
	G2	230	43.48
	G3	207	39.13
	G4	78	14.74
Stage	I	269	50.37
	II	57	10.67
	III	125	23.41
	IV	83	15.55
T stage	T1	275	51.21
	T2	69	12.85
	T3	182	33.89
	T4	11	2.05
M stage	M0	426	84.36
	M1	79	15.64
N stage	N0	240	93.39
	N1	17	6.61

### Enrichment Analysis of Differentially Expressed ARGs

We used a false discovery rate (FDR) <0.05 and a | log_2_ fold Change| (logFC) > 1 as screening criteria to obtain the differentially expressed ARGs. To better understand the role of differentially expressed ARGs, we used the “cluster Profiler” package (15) for enrichment analyses and then used the “GOplot package” (16) for visualization.

### Construction of the Autophagy-Related Prognostic Signature

We first obtained the prognostic ARGs in ccRCC by univariate Cox regression analysis and then performed multivariate Cox regression analysis and an optimized risk score model with the *step* function. The risk score was calculated as follows:

$$\begin{array}{l} \text{Risk score}=\sum_{i\;=\;1}^{\text{n}}\text{Coe}{\text{f}}_{\text{i}}\times x_{\text{i}} \end{array}$$

where Coef is the coefficient, and x is the expression value of each selected ARG. This formula was used to calculate the risk score for every ccRCC patient. Then we performed Cox regression analyses to demonstrate whether the autophagy-related signature was an independent prognostic factor in ccRCC patients.

### Gene Set Enrichment Analysis (GSEA)

Patients were divided into high- and low-risk groups according to the value of the risk scores. GSEA (17) was used to analyze which pathways genes are primarily enriched. GSEA was performed using GSEA3.0 (http://www.broad.mit.edu/gsea/). Differences for which the nominal *P* < 0.05 and the FDR < 0.25 were considered statistically significant.

### Statistical Analysis

Statistical analyses were performed with R software (Version 3.5.1). The Wilcox signed-rank test was used to compare the expression levels of differentially expressed ARGs between cancer tissues and normal tissues and the expression of 11 prognostic ARGs in the high- and low-risk groups. Student's *t*-test was used to compare the correlation between the risk score and clinicopathological variables. Cox regression analyses were used to screen genes for inclusion in the risk score model. ccRCC patients were divided into a high-risk and a low-risk groups according to the median value of the risk score, and OS and DFS of patients were analyzed by the Kaplan-Meier method and log-rank test. Receiver operating characteristic (ROC) curve analysis was performed with the “survivalROC” package. *P* < 0.05 was considered to be statistically significant.

## Results

### Differentially Expressed ARGs in Cancer Tissues and Normal Tissues

**Figure 1:**
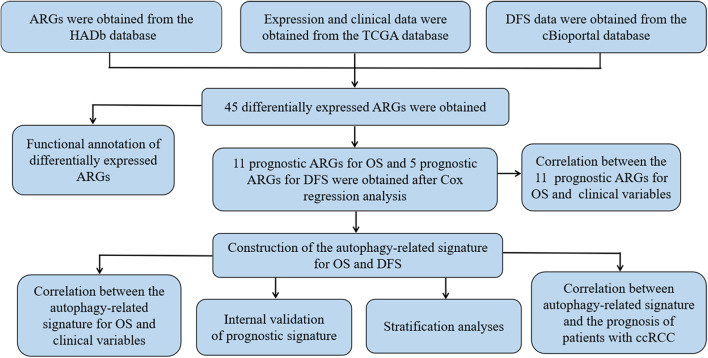
The flowchart of our research process.

**Figure 2:**
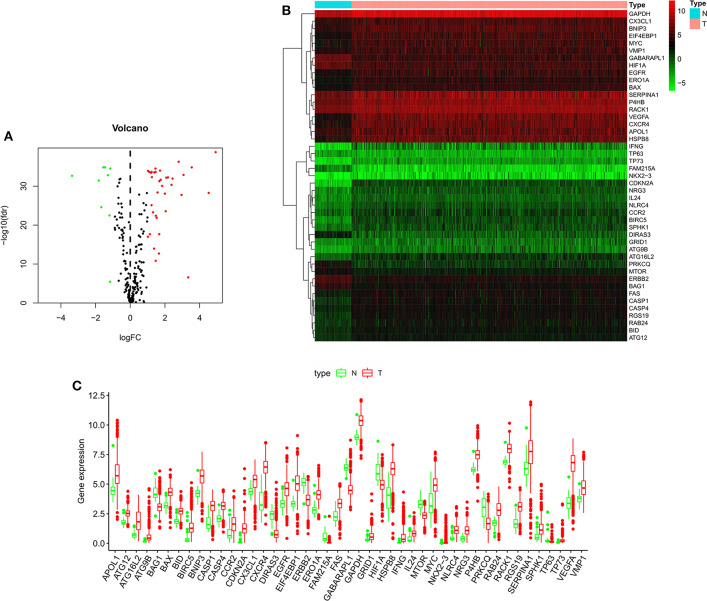
The expression of autophagy-related genes in ccRCC and normal kidney tissues. **(A)** Volcano plot of 222 autophagy-related genes. Red represents upregulated autophagy-related genes, green represents downregulated autophagy-related genes, and black represents autophagy-related genes with no difference in expression between ccRCC and normal kidney tissue. **(B)** Heatmap of the 45 differentially expressed autophagy-related genes. **(C)** Visualization of the expression levels of the 45 differentially expressed autophagy-related genes. Red represents tumor tissue, and green represents normal tissue. ccRCC, clear cell renal cell carcinoma.

Figure 1 shows the flow chart of our research process. We first obtained 232 ARGs from the HADb database and then downloaded the RNA-seq and clinical and prognostic data of 530 patients in the KIRC cohort from the TCGA database. We ultimately obtained the expression data of 222 ARGs. With FDR < 0.05 and | log_2_ FC| >1as the screening criteria, 45 differentially expressed ARGs were obtained, including 9 downregulated ARGs and 36 upregulated ARGs (Figures 2A,B). The expression of the differentially expressed ARGs between cancer tissues and normal tissues was visualized (Figure 2C). The following ARGs were upregulated: CX3CL1, ATG12, BID, IL24, RACK1, FAS, BAX, CASP4, VMP1, CCR2, P4HB, GAPDH, ERO1A, GRID1, EGFR, MYC, BNIP3, SERPINA1, SPHK1, RAB24, RGS19, CASP1, NLRC4, NRG3, APOL1, EIF4EBP1, HSPB8, ATG16L2, BIRC5, CXCR4, ATG9B, TP73, NKX2-3, VEGFA, IFNG and CDKN2A. The following ARGs were downregulated: FAM215A, DIRAS3, PRKCQ, GABARAPL1, ERBB2, BAG1, HIF1A, TP63 and MTOR.

### Functional Annotation of the Differentially Expressed ARGs

**Figure 3:**
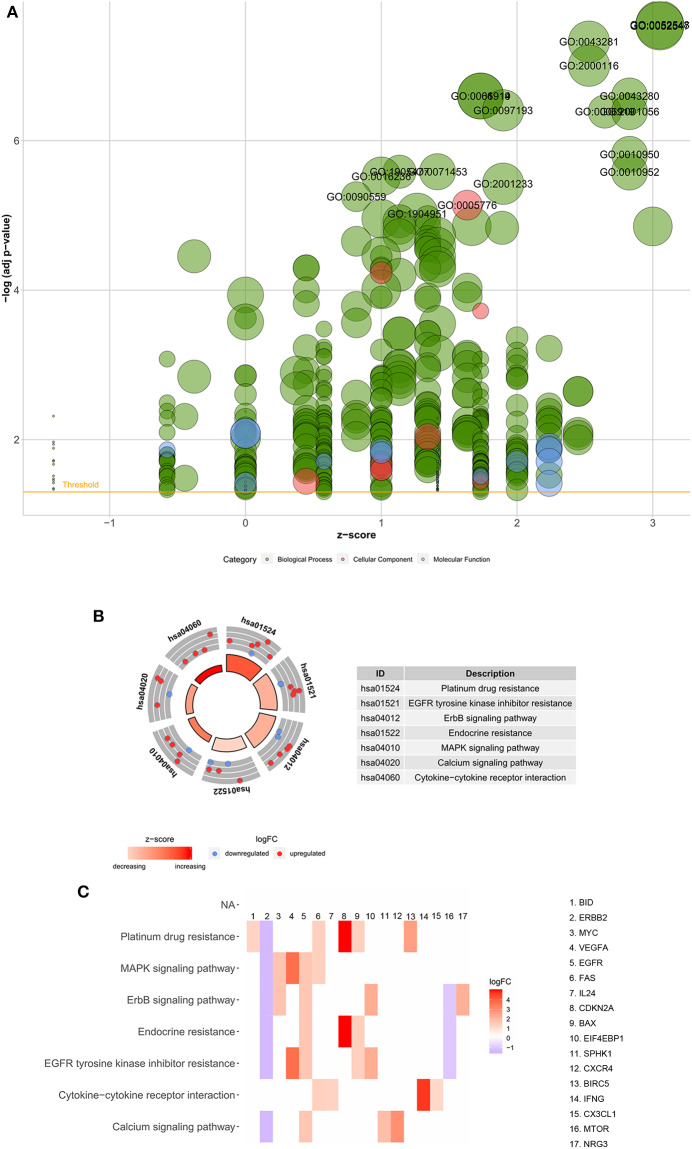
Functional enrichment analyses of the 45 differentially expressed autophagy-related genes. **(A)** Bubble diagram of enriched GO. The green circles represent biological processes, the red circles represent cellular components, and the blue circles represent molecular functions. **(B)** Circos plot of the KEGG pathway enrichment results. The inner red circle represents the z-score values, and the outer circle represents the number of genes enriched in the pathway. Red indicates upregulated autophagy-related genes, and green indicates downregulated autophagy-related genes. **(C)** Heatmap of the KEGG pathway enrichment results. Each bar represents a gene, and the depth of the bar represents the logFC value. GO, Gene Ontology; KEGG, Kyoto Encyclopedia of Genes and Genomes; FC, fold change.

Functional enrichment analysis was performed with the 45 differentially expressed ARGs. In the biological processes, the ARGs were mainly enriched in autophagy, positive regulation of peptidase activity, regulation of apoptotic signaling pathways, regulation of cell growth, autophagy of mitochondrion, autophagosome assembly, etc. In the cellular components, the ARGs were mainly enriched in autophagosomes, autophagosome membranes, cytosolic part, mitochondrial outer membrane, organelle outer membrane, vacuolar membrane, basal plasma membrane, etc. In the molecular functions, the ARGs were mainly enriched in protein phosphatase binding, P53 binding, phosphatase binding, chemokine receptor activity, cytokine activity, integrin binding, peptidase activator activity, receptor ligand activity, etc. (Table 2 and Figure 3A). In the Kyoto Encyclopedia of Genes and Genomes (KEGG) pathways, the ARGs were mainly enriched in platinum drug resistance, epidermal growth factor receptor (EGFR) tyrosine kinase inhibitor resistance, the epidermal growth factor receptor (ErbB) signaling pathway, endocrine resistance, the mitogen-activated protein kinase (MAPK) signaling pathway, calcium signaling pathway, and cytokine-cytokine receptor interaction (Table 2 and Figures 3B,C). The z scores of these KEGG pathways were >0, indicating that the ARGs were upregulated in these pathways.

**Table 2:** Functional enrichment analyses of the 45 differentially expressed autophagy-related genes.

**Category**	**ID**	**Term**	***P*-value**	**Genes**
Biological process	GO:0006914	Autophagy	7.70E-10	GABARAPL1, RAB24, ATG12, MTOR, GAPDH, IFNG, ATG16L2, RGS19, HIF1A, ATG9B, BNIP3, VMP1
Biological process	GO:0061919	Process utilizing autophagic mechanism	7.70E-10	GABARAPL1, RAB24, ATG12, MTOR, GAPDH, IFNG, ATG16L2, RGS19, HIF1A, ATG9B, BNIP3, VMP1
Biological process	GO:0097193	Intrinsic apoptotic signaling pathway	1.37E-09	P4HB, BID, TP63, RACK1, CASP4, ERO1A, BAX, TP73, HIF1A, BNIP3
Biological process	GO:0010952	Positive regulation of peptidase activity	1.60E-08	BID, RACK1, CASP4, NLRC4, FAS, BAX, MYC, CASP1
Biological process	GO:0016236	Macroautophagy	1.96E-08	GABARAPL1, ATG12, MTOR, GAPDH, ATG16L2, HIF1A, ATG9B, BNIP3, VMP1
Biological process	GO:2001233	Regulation of apoptotic signaling pathway	2.63E-08	P4HB, BID, TP63, RACK1, FAS, BAX, TP73, CX3CL1, HIF1A, BNIP3
Biological process	GO:2001235	Positive regulation of apoptotic signaling pathway	1.66E-07	BID, TP63, RACK1, FAS, BAX, TP73, BNIP3
biological process	GO:0001558	Regulation of cell growth	5.85E-07	VEGFA, CDKN2A, ERBB2, RACK1, PRKCQ, MTOR, EGFR, SPHK1, NRG3
Biological process	GO:0000422	Autophagy of mitochondrion	9.65E-07	GABARAPL1, ATG12, HIF1A, ATG9B, BNIP3
Biological process	GO:0000045	Autophagosome assembly	2.70E-06	GABARAPL1, ATG12, ATG16L2, ATG9B, VMP1
Cellular component	GO:0005776	Autophagosome	5.13E-08	GABARAPL1, RAB24, ATG12, ATG16L2, ATG9B, VMP1
Cellular component	GO:0000421	Autophagosome membrane	8.22E-07	GABARAPL1, ATG16L2, ATG9B, VMP1
Cellular component	GO:0044445	Cytosolic part	0.000258	RACK1, CASP4, NLRC4, MTOR, CASP1
Cellular component	GO:0005741	Mitochondrial outer membrane	0.000756	BID, MTOR, BAX, BNIP3
Cellular component	GO:0031968	Organelle outer membrane	0.001203	BID, MTOR, BAX, BNIP3
Cellular component	GO:0019867	Outer membrane	0.001247	BID, MTOR, BAX, BNIP3
Cellular component	GO:0005774	Vacuolar membrane	0.002060	GABARAPL1, MTOR, ATG16L2, ATG9B, VMP1
Cellular component	GO:0000407	Phagophore assembly site	0.036035	ATG12, ATG9B
Cellular component	GO:0009925	Basal plasma membrane	0.002743	ERBB2, EGFR
Cellular component	GO:0005793	Endoplasmic reticulum-Golgi intermediate compartment	0.002800	P4HB, VMP1, SERPINA1
Molecular function	GO:0019903	Protein phosphatase binding	0.000304	ERBB2, RACK1, EGFR, SPHK1
Molecular function	GO:0002039	P53 binding	0.000507	TP63, TP73, HIF1A
Molecular function	GO:0019902	Phosphatase binding	0.000911	ERBB2, RACK1, EGFR, SPHK1
Molecular function	GO:0004950	Chemokine receptor activity	0.001819	CCR2, CXCR4
Molecular function	GO:0005125	Cytokine activity	0.001955	VEGFA, IL24, IFNG, CX3CL1
Molecular function	GO:0004857	Enzyme inhibitor activity	0.002004	CDKN2A, BIRC5, RACK1, GAPDH, SERPINA1
Molecular function	GO:0005178	Integrin binding	0.003570	P4HB, EGFR, CX3CL1
Molecular function	GO:0016504	Peptidase activator activity	0.004065	RACK1,CASP1
Molecular function	GO:0048018	Receptor ligand activity	0.005909	VEGFA, IL24, IFNG, CX3CL1, NRG3
Molecular function	GO:0043022	Ribosome binding	0.007949	RACK1, MTOR
KEGG pathway	hsa01524	Platinum drug resistance	7.61E-07	CDKN2A, BID, ERBB2, BIRC5, FAS, BAX
KEGG pathway	hsa01521	EGFR tyrosine kinase inhibitor resistance	1.22E-06	VEGFA, ERBB2, MTOR, BAX, EGFR, EIF4EBP1
KEGG pathway	hsa04012	ErbB signaling pathway	1.89E-06	ERBB2, MTOR, MYC, EGFR, EIF4EBP1, NRG3
KEGG pathway	hsa01522	Endocrine resistance	7.87E-05	CDKN2A, ERBB2, MTOR, BAX, EGFR
KEGG pathway	hsa04010	MAPK signaling pathway	0.011471	VEGFA, ERBB2, FAS, MYC, EGFR
KEGG pathway	hsa04020	Calcium signaling pathway	0.012563	ERBB2, EGFR, CXCR4, SPHK1
KEGG pathway	hsa04060	Cytokine-cytokine receptor interaction	0.018991	IL24, FAS, IFNG, CX3CL1

*GO, Gene Ontology; KEGG, Kyoto Encyclopedia of Genes and Genomes*.

### Construction of a Prognostic Signature Based on the Prognostic ARGs for OS

**Figure 4:**
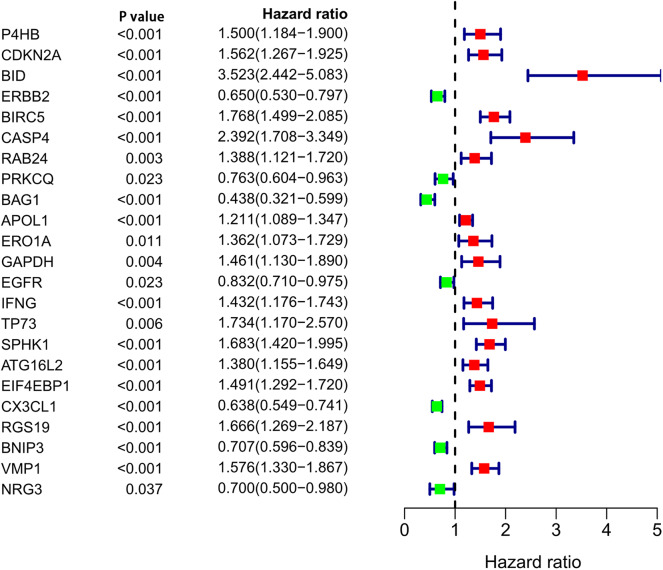
Univariate Cox regression analysis of differentially expressed autophagy-related genes.

After analyzing the expression and functions of the differentially expressed ARGs in ccRCC, we constructed a risk score model for the prediction of the prognosis of patients with ccRCC. After univariate Cox regression analysis, 23 ARGs were associated with the prognosis of ccRCC patients (Figure 4). After multivariate Cox regression analysis, 11 ARGs were identified and used to construct a prognostic signature for OS (Table 3). The risk score was calculated as follows: Risk score = (0.57 × *BID* expression) + (0.2696 × *ERBB2* expression) + (0.4565 × *CASP4* expression) + (0.2726 × *IFNG* expression) + (0.2433 × *ATG16L2* expression) + (0.2629 × *EIF4EBP1* expression) + (−0.4475 × *PRKCQ* expression) + (−0.3273 × *BAG1* expression) + (−0.2611 × *CX3CL1* expression) + (−0.4178 × *RGS19* expression) + (−0.3370 × *BNIP3* expression).

**Table 3:** Multivariate Cox regression analysis of prognostic autophagy-related genes.

**Gene**	**Coef**	**HR**	**95% CI**	**95% CI**
BID	0.57	1.768271	1.087791	2.874435
ERBB2	0.2696	1.309478	0.990010	1.732035
CASP4	0.4565	1.578542	1.015543	2.453656
PRKCQ	−0.4475	0.639218	0.486441	0.839979
BAG1	−0.3273	0.720856	0.496920	1.045709
IFNG	0.2726	1.313439	0.994634	1.734428
ATG16L2	0.2433	1.275483	1.058634	1.536749
EIF4EBP1	0.2629	1.300648	1.086314	1.557271
CX3CL1	−0.2611	0.770148	0.623699	0.95098
RGS19	−0.4178	0.658491	0.436949	0.992359
BNIP3	−0.3370	0.713904	0.573481	0.888710

*Coef, coefficient; HR, hazard ratio; CI, confidence interval*.

### The Correlation Between the Autophagy-Related Signature for OS and Prognosis of ccRCC Patients

**Figure 5:**
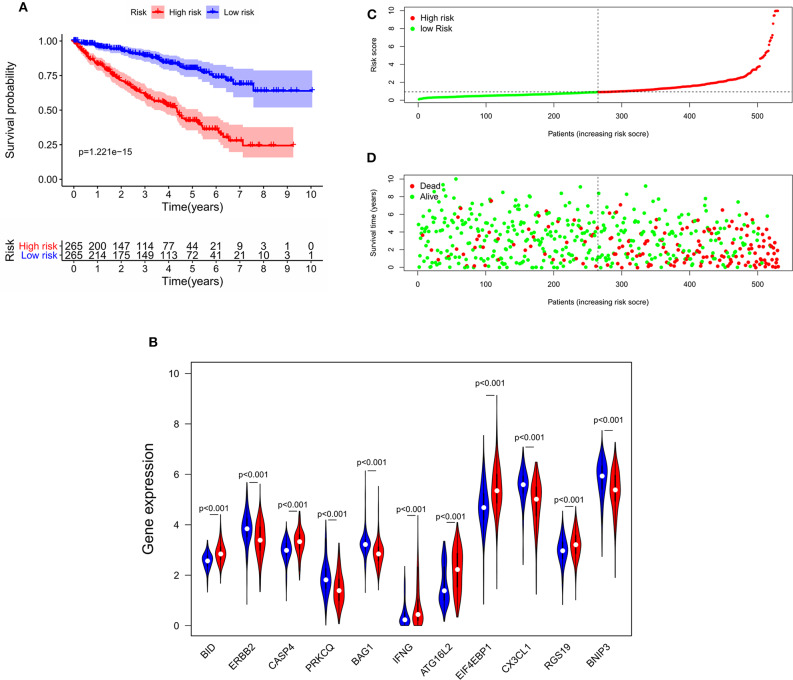
The correlation between the eleven-gene autophagy-related signature for OS and the prognosis of patients with ccRCC. **(A)** Kaplan-Meier OS curves for the high- and low-risk groups. **(B)** Expression of eleven autophagy-related genes in the high- and low-risk groups. Red represents the high-risk group, and blue represents the low-risk group. **(C)** Distribution of the risk scores of ccRCC patients. **(D)** The number of survivors and non-survivors with different risk scores; red represents the number of non-survivors, and green represents the number of survivors. OS, overall survival; ccRCC, clear cell renal cell carcinoma.

To determine the ability of the autophagy-related signature for OS to predict the prognosis of ccRCC patients, Kaplan-Meier analysis was performed to evaluate the OS outcomes in the two groups. The OS rate of patients in the high-risk group was significantly lower than that of patients in the low-risk group (*P* = 1.221e−15, Figure 5A), and the 5-years survival rates of patients in the high- and low-risk groups were 40.1 and 78.8%, respectively. The violin plot shows the expression of the eleven ARGs in the two groups. BID, RGS19, CASP4, IFNG, ATG16L2, and EIF4EBP1 were highly expressed in the high-risk group, and PRKCQ, BAG1, CX3CL1, ERBB2, and BNIP3 were highly expressed in the low risk group (Figure 5B). The risk score of patients in the high- and low- risk groups were visualized (Figure 5C). As the risk score increased, an increasing number of patients died (Figure 5D). These results showed that the risk score accurately reflect the survival of patients and that the autophagy-related signature for OS accurately predicts the prognosis of patients.

**Figure 6:**
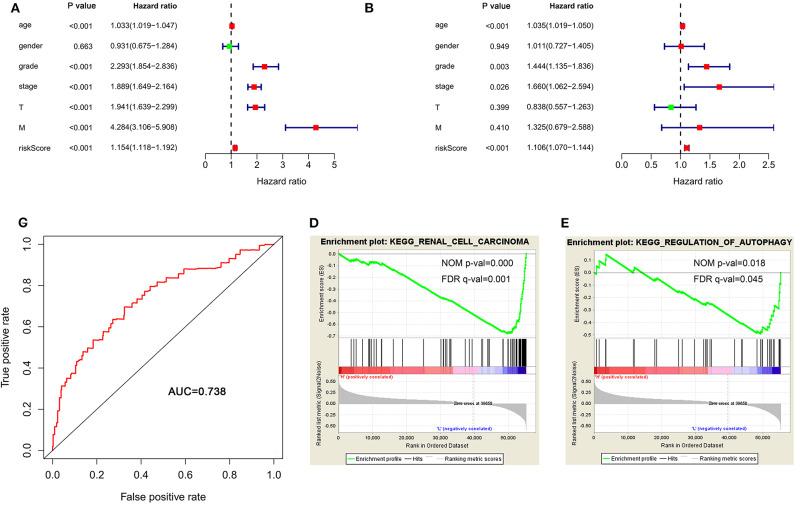
The autophagy-related signature for OS is an independent prognostic factor for ccRCC. **(A)** Univariate Cox regression analysis of correlations between the risk score for OS and clinical variables. **(B)** Multivariate Cox regression analysis of correlations between the risk score for OS and clinical variables. **(C)** ROC curve indicating the predictive accuracy of the autophagy-related signature for OS. **(D,E)** Gene set enrichment analysis comparing the high- and low-risk groups. ccRCC, clear cell renal cell carcinoma; ROC, receiver operating characteristic; OS, overall survival.

To determine whether the autophagy-related signature for OS is an independent prognostic factor for ccRCC patients, we performed Cox regression analysis. Univariate Cox regression analysis showed that age, stage, grade, T stage, M stage, and risk score were significantly associated with OS in ccRCC patients (Figure 6A). Multivariate Cox regression analysis showed that age, stage, grade and risk score were independent factors influencing ccRCC prognosis (Figure 6B). Then, a ROC curve was constructed to determine the predictive accuracy of the autophagy-related signature. The area under the curve (AUC) of the autophagy-related signature for OS was 0.738, indicating good predictive accuracy (Figure 6C).

Because of the survival differences between the high-risk and low-risk groups, we conducted GSEA to study the functional differences between these groups. The regulation of autophagy and renal cell carcinoma pathways were significantly enriched in the low-risk group (Figures 6D,E), indicating that the regulation of autophagy was mainly involved in low-risk ccRCC patients.

### Validation of Prognostic Signature Based on Prognostic ARGs for OS

**Figure 7:**
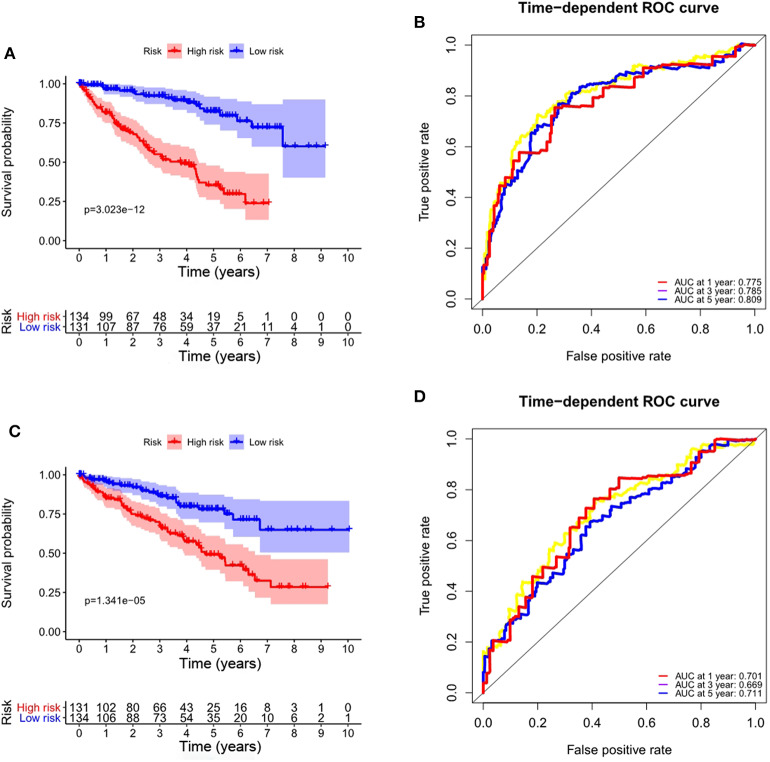
Validation of the prognostic signature based on prognostic ARGs for OS. **(A)** Kaplan-Meier OS curves for the high- and low-risk groups in the training set; **(B)** ROC curves in the training set; **(C)** Kaplan-Meier OS curves for the high- and low-risk groups in the validation set; **(D)** ROC curves in the validation set.

To validate the applicability of the prognostic signature for OS we constructed based on the entire TCGA data set, we randomly divided the 530 ccRCC patients in the entire data set into a training set (*n* = 265) and a validation set (*n* = 265). According to the formula, we calculate the risk score for each patient, and the patients in the training set and the validation set were divided into high- and low-risk groups based on the median value of the risk score. Consistent with the results observed in the entire data set, the OS rate of patients in the high-risk group was lower than that of the low-risk group in the training set (*P* = 3.023e-12, Figure 7A), and the prognosis of the high-risk group was worse than that of the low-risk group in the validation set (*P* = 1.341e-05, Figure 7C). The ROC curves of the training set and validation set also show good performance. The AUCs for the 1-year, 3-years, and 5-years OS of the training set were 0.775, 0.785, and 0.809, respectively (Figure 7B). The AUCs for the 1-year, 3-years and 5-years OS of the validation set were 0.701, 0.669, and 0.711, respectively (Figure 7D).

### Role of the Signature for OS in the Prognosis of ccRCC Patients Stratified by Clinicopathological Variables

**Figure 8:**
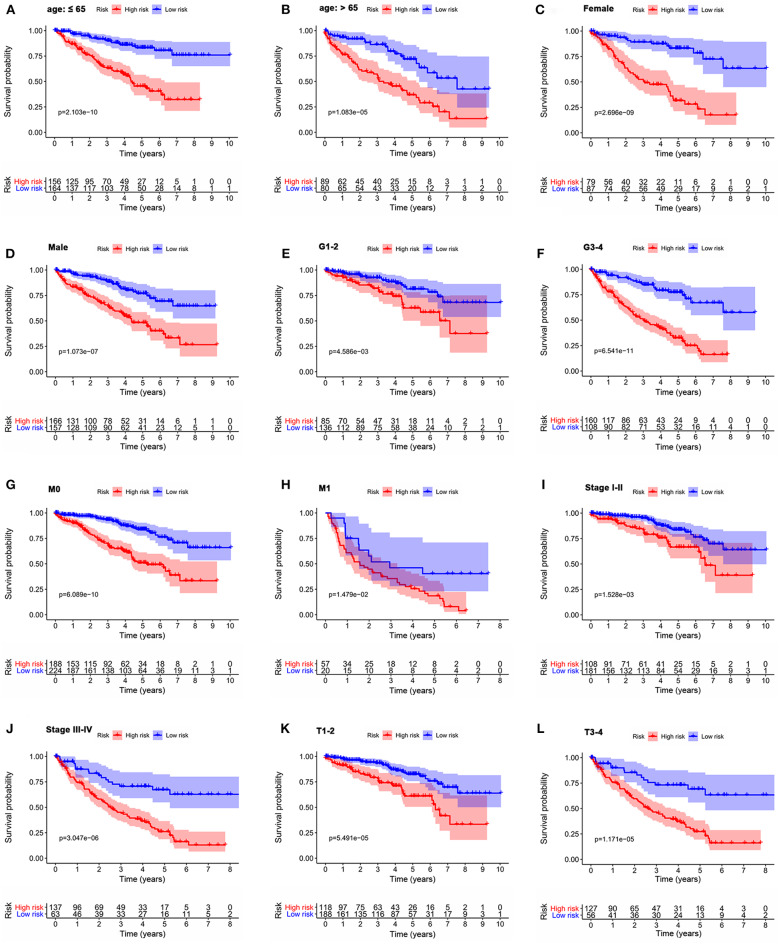
Kaplan-Meier survival curves for the high- and low-risk groups stratified by clinicopathological variables. **(A,B)** Age. **(C,D)** Gender. **(E,F)** Grade. **(G,H)** M stage. **(I,J)** Stage. **(K,L)** T stage. M, metastasis; T, tumor size.

To investigate the prognostic value of the signature for OS in ccRCC patients stratified by clinicopathological variables, ccRCC patients were stratified according to age, gender, grade, stage, M stage, and T stage. For all different stratifications, the OS time of the high-risk group was shorter than that of the low-risk group (Figure 8). These results suggest that the autophagy-related signature for OS can predict the prognosis of ccRCC patients without the need to consider clinicopathological variables.

### Relationship Between the Prognostic Signature for OS and Clinicopathological Variables

**Figure 9:**
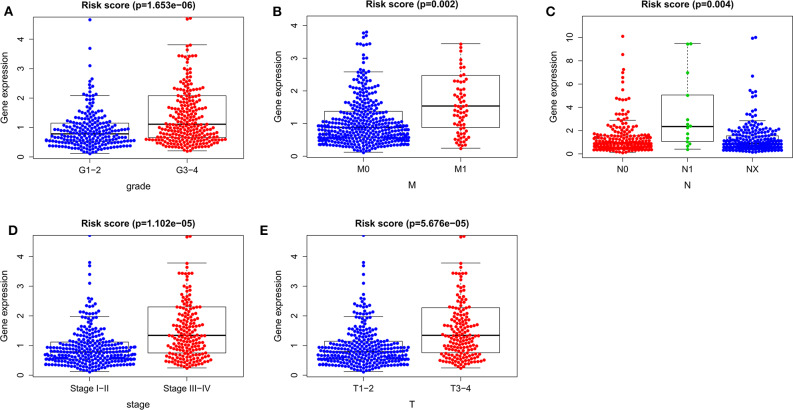
The relationships between the risk score and clinicopathological variables. **(A)** Grade. **(B)** M stage. **(C)** N stage. **(D)** Stage. **(E)** T stage. M, metastasis; T, tumor size; N, lymph node metastasis.

To determine whether the autophagy-related prognostic signature for OS affects the progression of ccRCC, we analyzed the correlations between the autophagy-related prognostic signature for OS and clinicopathological variables. The risk score of G3–4 was higher than that of G1–2 (*P* = 1.653e−06, Figure 9A), the risk score of M1 was higher than that of M0 (*P* = 0.002, Figure 9B), the risk score of N1 was higher than that of N0 (*P* = 0.004, Figure 9C), the risk score of stage III–IV was higher than that of stage I–II (*P* = 1.102e−05, Figure 9D), and the risk score of T3–4 was higher than that of T1–2 (*P* = 5.676e−05, Figure 9E). These results suggested that the higher the risk score is, the greater the degree of malignancy of ccRCC. Thus, the prognostic signature for OS could accurately predict the progression of ccRCC.

### Relationships Between the Prognostic ARGs and Clinicopathological Variables

To further understand the role of autophagy in ccRCC, we also studied the relationship between the prognostic ARGs for OS and clinicopathological variables. We found that BID, ERBB2, CASP4, PRKCQ, BAG1, INFG, EIF4EBP1, CX3CL1, RGS19 and BNIP3 were significantly associated with stage; BID, ERBB2, CASP4, BAG1, INFG, EIF4EBP1, CX3CL1, RGS19, and BNIP3 were significantly associated with grade; BID, ERBB2, CASP4, PRKCQ, BAG1, INFG, EIF4EBP1, CX3CL1, RGS19, and BNIP3 were significantly associated with T stage; BID, ERBB2, CASP4, BAG1, INFG, EIF4EBP1, and RGS19 were significantly associated with M stage; and ERBB2, PRKCQ, CX3CL1, and BNIP3 were significantly associated with gender. However, ATG16L2 had no significant correlation with gender, stage, grade, T stage or M stage (Table 4).

**Table 4:** The relationships between the prognostic ARGs and clinicopathological variables.

**Gene**		**Gender**		**Grade**		**Stage**		**T stage**		**M stage**	
		**Female**	**Male**	**G1-2**	**G3-4**	**I-II**	**III-IV**	**T1-T2**	**T3-T4**	**M0**	**M1**
N		166	323	221	268	289	200	306	183	412	77
BID	*t*-value	1.304		4.847		6.398		5.594		5.132	
	*P*-value	0.193		<0.001		<0.001		<0.001		<0.001	
ERBB2	*t*-value	2.474		4.432		5.506		5.760		2.769	
	*P*-value	0.014		<0.001		<0.001		<0.001		0.006	
CASP4	*t*-value	0.955		4.260		5.335		4.606		4.352	
	*P*-value	0.340		<0.001		<0.001		<0.001		<0.001	
PRKCQ	*t*-value	2.727		1.823		2.170		2.396		0.260	
	*P*-value	0.007		0.069		0.031		0.017		0.795	
BAG1	*t*-value	1.226		4.892		6.110		5.635		4.011	
	*P*-value	0.221		<0.001		<0.001		<0.001		<0.001	
INFG	*t*-value	0.526		4.781		5.255		4.579		4.713	
	*P*-value	0.599		<0.001		<0.001		<0.001		<0.001	
ATG16L2	*t*-value	1.837		0.203		1.467		1.607		0.507	
	*P*-value	0.067		0.839		0.143		0.109		0.612	
EIF4EBP1	*t*-value	0.508		5.484		6.521		5.897		4.502	
	*P*-value	0.611		<0.001		<0.001		<0.001		<0.001	
CX3CL1	*t*-value	3.803		4.427		3.964		4.404		1.663	
	*P*-value	<0.001		<0.001		<0.001		<0.001		0.097	
RGS19	*t*-value	1.168		6.124		5.055		4.237		2.942	
	*P*-value	0.244		<0.001		<0.001		<0.001		0.003	
BNIP3	*t*-value	2.446		3.045		1.969		2.273		0.634	
	*P*-value	0.015		0.003		0.049		0.023		0.526	

*ARGs, autophagy-related genes; T, tumor invasion; M, metastasis*.

### Construction of a Prognostic Signature Based on Prognostic ARGs for DFS

**Figure 10:**
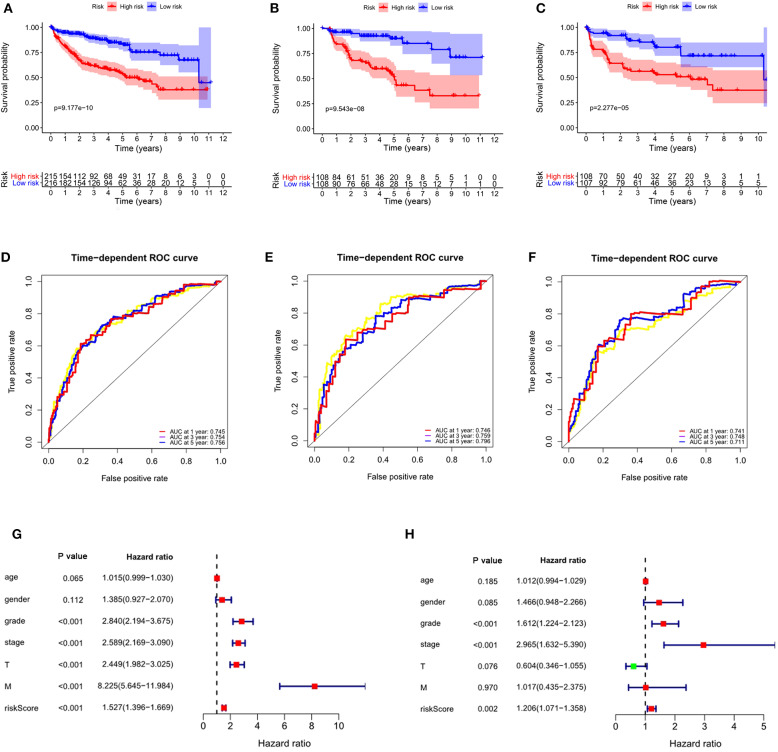
The autophagy-related signature for DFS is an independent prognostic factor for ccRCC. **(A)** Kaplan-Meier DFS curves for the high- and low-risk groups in the entire data set; **(B)** Kaplan-Meier DFS curves for the high- and low-risk groups in the training set; **(C)** Kaplan-Meier DFS curves for the high- and low-risk groups in the validation set; **(D)** ROC curves in the entire data set; **(E)** ROC curves in the training set; **(F)** ROC curves in the validation set; **(G)** Univariate Cox regression analysis of correlations between the risk score for DFS and clinical variables. **(H)** Multivariate Cox regression analysis of correlations between the risk score for DFS and clinical variables.

Considering the significance of DFS in the prognosis of ccRCC, we also established a prognostic signature for DFS. We obtained DFS data for ccRCC from cBioportal, including 431 patients. After univariate Cox regression analysis, we obtained 19 ARGs significantly correlated with DFS in ccRCC patients. After multivariate Cox regression analysis, we obtained 5 ARGs and constructed a prognostic signature: risk score = (0.5163 × *BID*) + (−0.4748 × *BAG1*) + (0.1084 × *APOL1*) + (−0.6522 × *NKX2-3*) + (0.3866 × *EIF4EBP1*). The risk score for each patient was calculated according to the formula, and the patients in the entire data set were divided into high- and low-risk groups according to the median value of risk score. K-M analysis showed that the DFS time of the high-risk group was significantly shorter than that of the low-risk group (*P* = 9.177e−10, Figure 10A), and ROC analysis showed that the AUCs for 1-year, 3-years, and 5-years DFS were 0.745, 0.754, and 0.756, respectively (Figure 10D). These results showed that the prognostic signature for DFS can also predict the prognosis of ccRCC patients well.

To verify the applicability of the prognostic signature for DFS, 431 patients were randomly divided into a training set (*n* = 216) and a validation set (*n* = 215). The risk score for each patient was calculated according to the formula, and the patients were divided into high- and low-risk groups according to the median value of risk score. Consistent with the results obtained from the entire data set, patients in the high-risk group in the training (*P* = 9.543e-08, Figure 10B) and validation sets (*P* = 2.277e-05, Figure 10C) had shorter DFS times than the low-risk groups. In the training set, the AUCs for 1-year, 3-years, and 5-years DFS were 0.746, 0.759, and 0.796, respectively (Figure 10E). In the validation set, the AUCs for 1-year, 3-years, and 5-years DFS were 0.741, 0.748, and 0.711, respectively (Figure 10F).

To determine whether the prognostic signature for DFS can independently predict the prognosis of ccRCC, we performed Cox regression analysis. Univariate Cox analysis showed that grade, stage, T stage, M stage and risk score were significantly correlated with DFS of ccRCC (Figure 10G). Multivariate Cox regression analysis showed that grade, stage and risk score were independent factors influencing the DFS of ccRCC (Figure 10H). These results showed that the autophagy-related signature for DFS could predict the DFS of patients well.

## Discussion

ccRCC is a disease that is likely to recur and to have a poor prognosis. Precise diagnostic and therapeutic biomarkers are urgently needed. Many studies have found that autophagy is significantly related to the occurrence and progression of cancer. However, current studies have focused on the influence of ARGs in cancer development and treatment (18–20), and few have addressed the prognostic value of ARGs in cancer. Recently, many studies have predicted the prognosis of ccRCC by constructing a prognostic signature based on miRNAs and lncRNAs (21–23), but few have reported the construction of an autophagy-related prognostic signature to predict the prognosis of ccRCC or a bioinformatic exploration of the possible role of ARGs in ccRCC. Although Wan et al. (24) constructed a prognostic model of autophagy-related genes for ccRCC with partial data sets from TCGA (*n* = 266). We used the entire TCGA data set (*n* = 530) for model construction. The differentially expressed ARGs in our two studies are also different, so our two constructed models are completely different. In our study, in addition to building the prognostic model for ccRCC, we also examined the role of ARGs in ccRCC.

First, we analyzed the differentially expressed ARGs in ccRCC and normal kidney tissues, and obtained 45 differentially expressed ARGs. GO and KEGG analyses indicated that the differentially expressed ARGs were mainly enriched mainly in platinum drug resistance. Studies have shown that the induction of autophagy in cancer can increase cisplatin resistance (25, 26), consistent with our results, suggesting that these ARGs can promote the progression of ccRCC through platinum drug resistance. However, further experiments are needed to verify the role of autophagy in ccRCC. Treating patients by inducing or inhibiting autophagy remain controversial (27). The overexpression of ARGs in lung cancer tissues can promote the progression of lung cancer (28). ARG Beclin-1 is highly expressed in colorectal cancer, and Beclin-1 high expression is positively correlated with clinicopathological variables and predicts good prognosis (29). However, some studies have found that Beclin-1 is expressed at low levels in bladder cancer (30), salivary gland adenoid cystic carcinoma (31), and pancreatic ductal adenocarcinoma (32) and that patients with low Beclin-1 expression have shorter survival times. ARGs are expressed differently in different cancers, possibly due to tumor heterogeneity.

Autophagy is closely related to the prognosis of cancer patients, so it is important to find a prognostic signature for ccRCC patients. We used univariate Cox regression to analyze ARGs associated with the prognosis of patients with ccRCC. 23 ARGs were found to be significantly associated with the prognosis of ccRCC. Then, multivariate Cox regression analysis was performed, and 11 ARGs (BID, ERBB2, CASP4, PRKCQ, BAG1, IFNG, ATG16L2, EIF4EBP1, CX3CL1, RGS19, and BNIP3) were identified for inclusion in the risk score model for OS. *In vivo* and *in vitro* experiments showed that knockdown of CASP4 leads to cell migration and impairs cell-matrix adhesion (33). Silencing BAG1 in breast cancer cells increases resistance to tamoxifen and reduces apoptosis by activating the PI3K/Akt/mTOR signaling pathway (34), and the overexpression of ATG16L2 is related to poor prognosis in epithelial cancer (35). The overexpression of EIF4EBP1 is related to shorter recurrence-free survival in breast cancer patients (36). CX3CL1 is highly expressed in esophageal cancer and can promote its metastasis (37). RGS19 can effectively inhibit Ras-related carcinogenesis in lung cancer (38). BNIP3 has an anticancer effect and is negatively correlated with the expression of the m6A demethylase FTO in breast cancer; BNIP3 can slow down the growth and metastasis of FTO-overexpressing tumors (39). Our GSEA results showed that the regulation of autophagy was mainly enriched in the low-risk group, indicating that autophagy has a greater regulatory role and influence in the low-risk group than in the high-risk group. However, whether autophagy is negatively regulated in the low-risk group requires further study. The next study demonstrated that the autophagy-related signature for OS can independently predict the prognosis of ccRCC patients and is a good predictor of ccRCC patient prognosis. The higher the risk score was, the worse the prognosis and the greater the degree of malignancy. Through the internal validation with the training set and the validation set, the autophagy-related signature for OS we constructed was shown to have good predictive performance.

We also found that the autophagy-related signature for OS can predict the prognosis of ccRCC patients without the need to consider clinicopathological variables. In addition, we also established a prognostic signature for DFS and conducted internal validation. The autophagy-related signature for DFS can independently and accurately predict the prognosis of ccRCC patients. However, our research also has some limitations. First, the mechanisms of action of the ARGs in ccRCC need validation *in vivo* and *in vitro* experiments. Second, we only used data from the TCGA database for this analysis, and we should validate the results in other databases. Although we have performed internal verification, we still need to perform external validation in other cohorts to test the applicability of the autophagy-related signature.

In conclusion, we constructed the autophagy-related signature for OS and DFS that can independently predict the prognosis of ccRCC patients and provide new therapeutic targets for ccRCC. We have developed a deep understanding of the biological mechanisms and clinical significance of the identified ARGs in ccRCC, but further experiments are still needed to verify our findings in the future.

## Data Availability Statement

The datasets analyzed for this study can be found in the Human Autophagy Database (http://www.autophagy.lu/), The Cancer Genome Atlas (https://portal.gdc.cancer.gov/) and cBioportal (https://www.cbioportal.org/).

## Author Contributions

MC and SZ contributed conception and design of the study. MC, ZN, and XW organized the database. XW, SZ, and YG performed the statistical analysis. MC wrote the first draft of the manuscript. SZ and YG wrote sections of the manuscript. All authors contributed to manuscript revision, read and approved the submitted version.

## Conflict of Interest

The authors declare that the research was conducted in the absence of any commercial or financial relationships that could be construed as a potential conflict of interest.
